# Blood pro-oxidant/antioxidant balance in young men with class II obesity after 20 sessions of whole body cryostimulation: a preliminary study

**DOI:** 10.1080/13510002.2021.1881328

**Published:** 2021-02-09

**Authors:** Wanda Pilch, Joanna Wyrostek, Anna Piotrowska, Olga Czerwińska-Ledwig, Roxana Zuziak, Ewa Sadowska-Krępa, Marcin Maciejczyk, Małgorzata Żychowska

**Affiliations:** a Institute for Basics Sciences, Faculty of Physiotherapy, University of Physical Education, Krakow, Poland; b University of Physical Education, Krakow, Poland; c Institute of Sport Sciences, The Jerzy Kukuczka Academy of Physical Education, Katowice, Poland; d Institute for Biomedical Sciences, Faculty of Physical Activity and Sport, University of Physical Education, Krakow, Poland; e Department of Sport, Faculty of Physical Education, Kazimierz Wielki University, Bydgoszcz, Poland

**Keywords:** Obesity management, cryotherapy, cryostimulation, oxidative stress index (OSI), prooxidant-antioxidant status (PAS), antioxidative enzymes, pro-oxidant-antioxidant balance (PAB)

## Abstract

**Objectives:** In obesity, there is a shift in the pro-oxidative-antioxidant balance towards the oxidationreactions. However, it has been shown that in people with normal body composition, after a series of whole-body cryotherapy (WBC), the balance shifts in the opposite direction.

**Design:** The aim of the study was to assess the impact of 20 WBC treatments on blood pro-oxidative-antioxidant balance.

**Interventions:** Study included 14 obese (BMI > 35) and 10 non-obese volunteers.

**Methods:** The total antioxidative (TAS/TAC) and pro-oxidative status (TOS/TOC) in serum and activity of antioxidant enzymes in erythrocytes were determined before the first and 2 hours after the last cryostimulation.

**Results:** In the obese group, a significantly higher level of TOS/TOC, and its significant decrease after the WBC series, was observed. Cryotherapy had no influence on TAS/TAC level which was similar in both groups. Changes in activity of antioxidant enzymes were multidirectional. An increase in CAT activity in the obese group was observed. OSI, both before and after a series of treatments, was significantly higher in obese subjects.

**Conclusions:** A beneficial effect on the level of TOS/TOC and CAT activity was indicated, but the proposed number of treatments for patients with class II obesity turned out to be insufficient.

**Trial registration:** Australian New Zealand Clinical Trials Registry identifier: ACTRN12619000524190.

## Introduction

According to epidemiological data, being overweight or obese is now a global health problem, occurring in various populations, and as a result, is becoming a serious clinical and economic challenge for healthcare systems around the world [1,2]. Obesity is a chronic disease that may be influenced by genetic, environmental, psychological, metabolic and endocrine factors, in addition to being affected by certain medications. It is well known to be associated with chronic inflammation, which is characterized by overproduction of inflammatory cytokines and acute phase proteins, that induce oxidative stress [3]. Earlier studies have revealed increased oxidative stress parameters in obese people. Increased levels of markers of oxidative stress such as malondialdehyde (MDA), thiobarbituric acid reactive species (TBARS) and 8-epi-prostaglandin F2α (8-epi-PGF2α) have been observed in obesity [4–6].

It is also suggested that the production of reactive oxygen and nitrogen species (RONS) in adipose tissue may be stimulated by the secretion of adipokines [3], including leptin [7]. It has also been found that the increased concentration of free fatty acids leads to mitochondrial uncoupling and increased generation of ROS [8]. RONS may affect the apoptosis of Langerhans cells in the pancreas, disrupting glucose-insulin homeostasis, while hyperglycemia leads to a further increase in oxidative stress [8]. The result of these processes may be increasing insulin resistance, leading to development of type II diabetes, which is a common complication of obesity. Nevertheless, the link between pathomechanisms of obesity and oxidative stress remains unclear.

It has been documented that whole body cryotherapy (WBC) treatments have anti-inflammatory effects, resulting in reduced levels of oxidative stress [9–13]. Cold therapy (whole body cryotherapy and partial body cryotherapy) is used to relieve pain and eliminate inflammation. It has a number of physiological and psychological benefits in humans, allowing cryotherapy to be used in medicine, wellness and sports. The standard procedure prescribed by rheumatologists, traumatologists or sports physicians includes a series of 20 WBC treatments [14]. A single treatment causes disruption of the prooxidative-antioxidant balance (PAB), with a decrease in total oxidative status (TOS) and total antioxidative status (TAS) of plasma, with their subsequent increase the next day after WBC treatment [9]. In turn, repetition of WBC treatments can cause adaptive changes in the human body in the form of increased activity of antioxidant enzymes and plasma antioxidant capacity, as well as a reduction in the concentration of oxidative stress markers (conjugated dienes and MDA). These changes have a positive effect on maintaining a higher level of PAB. In this way, there may be an increase in the anti-inflammatory properties of systemically used cryogenic temperatures and their protective effect against oxidative stress [9,10].

According to a review of the literature, obese people have a shift in the pro-oxidant–antioxidant balance towards the oxidation reactions, and after a series of cryotherapy treatments, there is a shift in balance in the opposite direction. Despite the fact that our subjects had class II obesity, we predicted that there will be an increase in antioxidant capacity with a simultaneous decrease in pro-oxidative capacity in this case as well. Therefore, the main goal of the study was to assess the impact of 20 WBC treatments on blood pro-oxidative-antioxidant balance in young obese men. To assess this, the activity of selected antioxidative enzymes, i.e. superoxide dismutase (SOD1), catalase (CAT) and glutathione peroxidase (GPx), total antioxidative potential (TAS/TAC) and pro-oxidative potential (TOS/TOC) of plasma after a series of 20 systemic cryostimulation treatments were analyzed in this study.

## Materials and methods

### Study group

**Figure 1.**
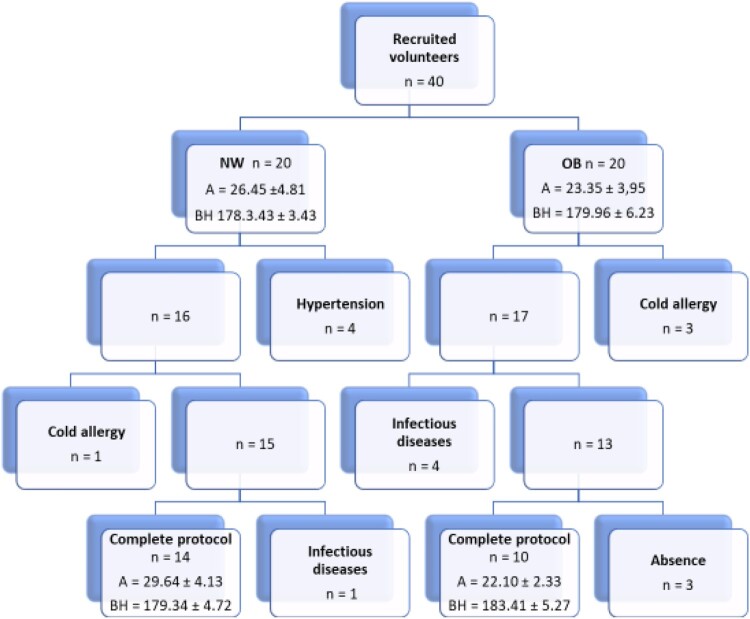
Patient flow diagram. A – age, BH – body height, NW – volunteers with normal BMI, OB – volunteers with BMI> 35.

The study involved 40 volunteers who were interviewed and had a medical examination to exclude any contraindications for cryostimulation. The inclusion criteria consisted of: BMI in the normal range (NW, normal weight group) and BMI over 35 (OB, obese group), age 20–35 years, male gender, no contraindications for systemic cryostimulation procedures and consent by the qualifying doctor to participate in the study. Exclusion criteria were: taking anti-inflammatory drugs and supplements containing vitamins and antioxidants. Glycemic levels allowing the diagnosis of diabetes were also one of exclusion criterium. Each procedure was preceded by blood pressure control (threshold value preventing the continuation of participation in the project: 160/100 mmHg). Respondents were asked to maintain their nutrition habits and recreational physical activity levels. A full protocol was completed by 24 young men (Figure 1): 14 with high and 10 with normal body weight. Study group characteristics are shown in Table1. The reasons for falling out of participants were: high blood pressure before entering cryochamber, respiratory tract infections, cold intolerance, resignation without giving any reason.

**Table 1.** Anthropometric characteristics of the subjects.

Parameter	OB (*n* = 14) $\bar{x}$ ± SD	NW (*n* = 10) $\bar{x}$ ± _SD_
A [years]	29.64 ± 4.13	22.10 ± 2.33#
BH [cm]	179.34 ± 4.72	183.41 ± 5.27
BM [kg]	115.61 ± 25.83	79.30 ± 8.44#
BMI [kg·m^−2^]	35.88 ± 0.53	23.56 ± 1.89#
PBF [%]	31.73 ± 5.90	12.27 ± 4.67#

NW – volunteers with normal BMI, OB – volunteers with BMI> 35, A – age, BH – body height, BM – body mass, BMI – body mass index, PBF – percentage of body fat; #statistically significant differences between studied groups (*p *< 0.05)

The respondents, in accordance with the Declaration of Helsinki, were informed about the purpose and method of research, the possibility of resignation at each stage of the project without any consequences, provided insight into their results and gave their written consent to participate in the study. The consent of the Bioethics Committee at the Regional Medical Chamber in Krakow (number 122/KBL/OIL/2017) was obtained for conducting this research. This project obtained the trial registered number on ANZCTR (ACTRN12619000524190, date: 2.04.2019).

### Systemic cryostimulation procedure

The study participants were subjected to 20 sessions of systemic cryostimulation (1 treatment per day, from Monday to Friday, excluding public holidays) at −120°C for 2–3 min, in a cryochamber located at the Malopolska Cryotherapy Center in Krakow. In order to prepare the body for extremely low temperatures, each cryostimulation procedure was preceded by a 30-second period of adaptation in the atrium of the cryochamber at a temperature of −60°C. Treatments took place under the supervision of a trained employee of the facility. The examined men were dressed in shorts, mid-calf socks, clogs, gloves and a cap covering the ears. The nose and mouth were covered with a surgical mask. Project participants were instructed on the need for constant movement and slow breathing (short breath, long breath). Continuous contact with the study group was maintained by a camera and voice system. To check cardiovascular response to cold temperatures, each participant had a measurement of systolic and diastolic blood pressure before and after the procedure. An increased pressure above the limit value of 160/100mmHg after the WBC procedure eliminated the patient from further participation in the project. The subjects were not subjected to any form of kinesitherapy after the cryotherapy procedure.

### Blood collection and biochemical analyses

Blood was collected for testing up to two hours before the start of first systemic cryostimulation and up to two hours after the end of a series of 20 treatments. Collections were performed according to current recommendations by a laboratory diagnostician using a Vacutainer vacuum system (Becton Dickinson, USA) for tubes with coagulation activator and tubes containing EDTA as an anticoagulant. Serum was obtained from the blood collected in a tube containing clot activator after centrifugation (MPW 350R laboratory centrifuge, MPW Med Instruments, Poland, centrifugation parameters: 10 min, 2500 rpm, temperature: 4°C). All stages of serum preparation were conducted in an acrylic glovebox station for anaerobic work (MBRAUN, MB GB 2202, Germany) with the use of nitrogen (Linde Gaz, Poland) in order to prevent oxidation of sample components. Suspension of erythrocytes was obtained from the collected blood by the addition of EDTA, centrifugation of the sample (centrifugation parameters: 10 min, 2500 rpm, temperature: 4°C), plasma extraction and then washed 3 times with 0.9% sodium chloride solution and centrifuged (discarding the supernatant each time). Both serum and suspension of erythrocytes were stored frozen at −80°C until analysis (no longer than 3 months).

### Biochemical analyses

## Total plasma antioxidant status

Total antioxidant status/capacity (TAS/TAC) was assessed with use of photometric kit (Immunodiagnostik AG, Bensheim, Germany). Total oxidant status/capacity (TOS/TOC) determination was made by using the Immunodiagnostik AG (Bensheim, Germany) test. Measurements were made using a Chromate 4300 Microplate Reader (Awarness Technology, USA) at a wavelength of 450 nm, relative to a calibrator. The obtained optical density (OD) values for individual samples were calculated according to the formulas indicated by the test manufacturer. Oxidative stress index (OSI) was defined as the percentage ratio of TOC levels to TAC levels [15].

## Antioxidant enzymes

A heparinized blood was centrifuged for 10 min at 1000× g at 4°C to separate erythrocytes that were then washed three times with cold saline (4°C) and kept frozen at −80°C until analysis superoxide dismutase (SOD1) using the commercially available RANSOD SD125 kit (Randox Laboratories Ltd., Crumlin, UK); glutathione peroxidase (GPx) with the commercial RANSEL RS505 kit (Randox Laboratories Ltd., Crumlin, UK) and catalase (CAT, EC 1.11.1.6) by the method of Aebi [16]. The activities of all antioxidant enzymes were measured at 37°C and expressed per 1 g of hemoglobin as assayed by a standard cyanmethemoglobin method using a diagnostic kit No. HG980 (Randox Laboratories Ltd., Crumlin, UK).

## Statistical analysis

For all parameters, the type of distribution was checked using the Shapiro–Wilk normality test. To determine the *p* value within groups, the *t*-test for dependent means was applied, while between groups, unpaired t-test was used. Level of significance was set at *p* ˂ 0.05. For confirmation of differences between groups, 2-way ANOVA was applied. For calculation of OSI and CAT/SOD1 ratio, quotients were calculated. Pearson’s correlation was used to analyze relationships between age and obtained data. All statistical analyses were performed using Prism 6.0 (GraphPad).

## Results

### Measures of TAS/TAC, TOS/TOC and OSI

**Figure 2.**
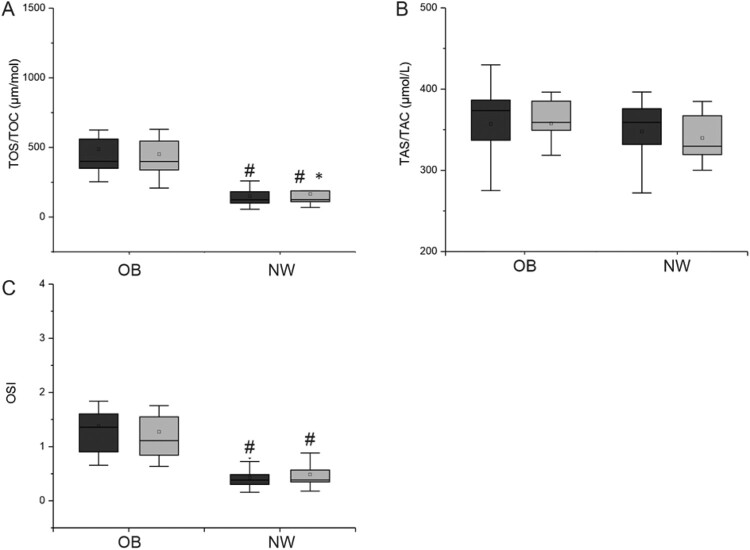
Comparison of TOS/TOC and TAS/TAC results in study groups. A – TOS/TOC [umol/l], B – TAS/TAC [umol/l], C – OSI. Dark bars – blood collection before first participation in whole body cryotherapy procedure, gray bars – blood collection after a series of 20 treatments. Statistically significant changes (*p* < 0.05): # OB vs. NW, * baseline vs. after 20 whole body cryotherapies, NW – volunteers with normal BMI, OB – volunteers with BMI> 35.

Changes in TAS/TAC, TOS/TOC and OSI in both groups are presented in Figure 2 and in Table 2.

**Table 2.** Summary means and SD values of the measured parameters.

Parameter	Group	I	II	*p*	2-way ANOVA
TOS/TOC [umol/L]	NW	150.30 ± 81.29	165.8 ± 92.57	0.04*	*interaction, row factor, time
	OB	487.78 ± 220.77	451.57 ± 206.47	0.12	
	*p*1 (NW I vs OB I) = 0.0001#; *p*2 (NW II vs OB II) = 0.0003#				
TAS/TAC [umol/L]	NW	347.6 ± 39.09	339.9 ± 29.15	0.62	*row factor
	OB	357.14 ± 45.56	357.64 ± 43.11	0.94	
	p1 (NW I vs OB I) = 0.40 p2 (NW II vs OB II) = 0.23				
OSI	NW	0.43 ± 0.20	0.49 ± 0.26	0.08	*interaction, row factor, time
	OB	1.47 ± 0.69	1.27 ± 0.59	0.16	
	p1 (NW I vs OB I) = 0.0001#; p2(NW II vs OB II) = 0.0008#				
CAT [µg/Hb]	NW	188.06 ± 25.31	189.30 ± 28.34	0.88	*interaction, time
	OB	195.85 ± 25.15	220.90 ± 39.40	0.01*	
	p1 (NW I vs OB I) = 0.46; p2 (NW II vs OB II) = 0.04#				
SOD1 [µg/Hb]	NW	1460.83 ± 262.68	1261.85 ± 132.51	0.03*	ns.
	OB	1226.42 ± 136.19	1384.28 ± 227.48	0.04*	
	p1 (NW I vs OB I) = 0.01#; p2 (NW II vs OB II) = 0.14				
GPx [µg/Hb]	NW	25.38 ± 1.75	24.67 ± 2.15	0.52	ns.
	OB	26.32 ± 4.43	28.04 ± 4.74	0.37	
	p1 (NW I vs OB I) = 0.53; p2 (NW II vs OB II) = 0.04#				
CAT/SOD1 Ratio	NW	0.13 ± 0.03	0.15 ± 0.03	0.10	*interaction, time
	OB	0.16 ± 0.02	0.16 ± 0.04	0.84	
	p1 (NW I vs OB I) = 0.02#; p2 (NW II vs OB II) = 0.47				

NW – volunteers with normal BMI, OB – volunteers with BMI> 35, I – data obtained before the experiment; II – data obtained after 20th cryotherapy.

Initially, a significantly higher concentration of lipid peroxidation products was observed in obese men (*p* < 0.0001). Participation in 20 cryostimulation treatments significantly reduced PerOX to only in the NW group (Figure 2(A)). Such changes were not observed in the OB group. These results were confirmed in 2-way ANOVA which showed significancy for row factor and effect of time. Serum antioxidant capacity did not significantly differ between subjects from both groups before and after the series of systemic cryostimulations (Figure 2(B)). The change in the OB group was insignificant (*p* > 0.05). However, 2-way ANOVA was significant for row factor.

The ratio of peroxidated lipids to antioxidant capacity (OSI) was significantly higher in the group of obese subjects in comparison with NW group during the whole experiment (before *p* < 0.0001 and after *p* < 0.0008). However, thermal stimulus didn’t affect OSI levels in study groups (Figure 2(C)).

### Changes in antioxidative enzymes activities

**Figure 3.**
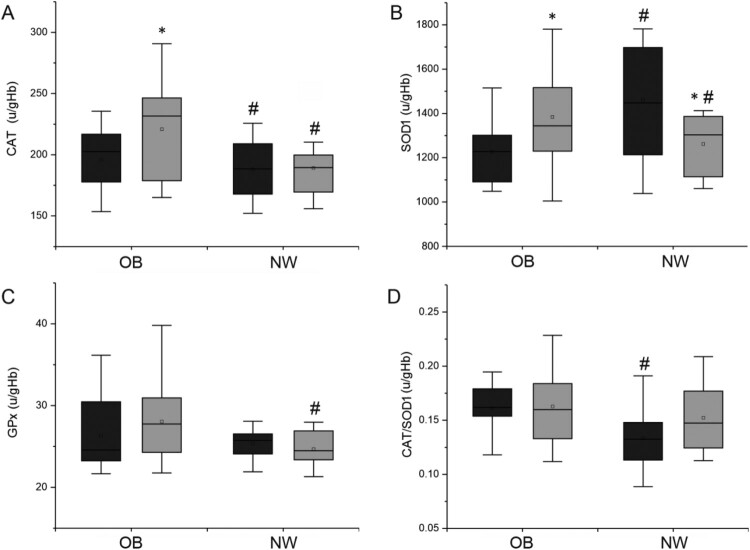
Antioxidant enzymes activity in erythrocytes per gram of hemoglobin in study groups. A – CAT, catalase [u/gHb], B – SOD1, superoxide dismutase [u/gHb], C – GPx, glutathione peroxidase [u/gHb], D – CAT/SOD1 ratio. Dark bars – blood collection up to 2 h before participation in first whole body cryotherapy procedure, gray bars – blood collection up to 2 h after a series of 20 treatments. Statistically significant changes (*p* < 0.05): # OB vs. NW group, * baseline vs. after 20 whole body cryotherapies, NW – volunteers with normal BMI, OB – volunteers with BMI > 35.

Figure 3 shows changes in the activity of antioxidant enzymes.

Changes in antioxidant enzymes activity were multidirectional. Twenty cryostimulation treatments caused a significant increase in CAT activity in the OB group (*p *= 0.01). After 20 cryostimulation sessions, differences in activity of this enzyme between groups were also significant (*p *= 0.04). 2-way ANOVA showed significant differences for the effect of time and interaction between group and time (Figure 3(A)). SOD1 activity was significantly higher in NW before the experiment (*p* = 0.01). Twenty cryostimulations caused a significant increase in SOD1 activity in the OB group (*p *= 0.04) and a significant decrease in the NW group (*p *= 0.03). These significant differences were not confirmed in 2-way ANOVA. After 20 cryostimulation treatments GPx activity changed insignificantly – decreased in NW group and increased in OB group. However, analysis of the results between groups obtained after 20 sessions showed that the thermal stimulus significantly affected activity of this enzyme (*p* = 0.04). These significant differences were not confirmed in 2-way ANOVA.

These multidirectional changes in antioxidative enzymes caused a change in CAT/SOD1 ratio. During the experiment, an increase in CAT/SOD1 ratio was observed in the NW group, while in the OB group, this indicator remains unchanged. While there were significant differences between groups at baseline (*p* = 0.02), at the end of the experiment, the values ware similar in both groups. However, significant interaction between time and group and the effect of time were confirmed by 2-way ANOVA.

Correlation analysis allowed to indicate a weak relationship between the change in SOD1 activity and the change in GPx activity for all subjects (*r* = 0.489 *p* = 0.015). In the OB group, a positive correlation was indicated between the baseline SOD1 and GPx activities (*r* = 0.702 *p* = 0.005) and between the change in SOD1 (ΔSOD) activity and the change in GPx activity (ΔGPx) (*r* = 0.556 *p* = 0.039). In NW group, an inverse correlation was indicated between TAS/TAC and the initial level of SOD1 enzyme activity (*r* = −0.699 *p* = 0.025) and between the change in CAT activity (ΔCAT) and change in GPx activity (ΔGPx) (*r* = −0.849 *p* = 0.002).

### Relationships between age and measured parameters

As the OB and NW groups differed significantly in age, the relationship between age and the measured PAB indicators was analyzed. The results of this analysis are presented in Table 3. Statistically significant correlations obtained for all subjects were noted for the SOD1 activity (*r* = −0.421 *p* = 0.040) and the baseline TAS/TAC values (*r* = 0.419 *p* = 0.042). The relationship between GPx activity measured after a series of treatments and the age of the subjects (*r* = 0.641 *p* <0.001) and the size of the obtained differences for the activity of this enzyme (*r* = 0.416 *p* = 0.043) was also indicated. In the group of obese patients, a weak influence of age on GPx activity after a series of treatments in the cryochamber was noted (*r* = 0.583 *p* = 0.029). In people with normal BMI, the only statistically significant relationship with age was obtained for GPx (baseline value and ΔGPx). The relationship of baseline activity of this enzyme in the NW group was the strongest observed correlation (*r* = −0.723 *p* = 0.018).

**Table 3.** Relationships between age and measured parameters.

	Total		OB		NW	
	*r*	*p*	*r*	*p*	*r*	*p*
TAS/TAC I	0.419*	0.042	0.485	0.079	0.094	0.797
TAS/TAC II	0.139	0.518	−0.056	0.849	−0.043	0.906
ΔTAS/TAC	−0.255	0.229	−0.481	0.082	−0.103	0.777
TOS/TOC I	−0.117	0.586	−0.167	0.568	−0.352	0.319
TOS/TOC II	−0.081	0.707	−0.108	0.713	−0.619	0.056
ΔTOS/TOC	0.057	0.790	0.086	0.770	−0.394	0.260
CAT I	0.169	0.430	−0.050	0.865	0.417	0.230
CAT II	0.321	0.127	0.033	0.912	−0.043	0.906
ΔCAT	0.251	0.237	0.077	0.793	−0.465	0.175
SOD1 I	−0.421*	0.040	−0.087	0.768	−0.052	0.886
SOD1 II	0.087	0.687	−0.220	0.450	−0.239	0.506
ΔSOD1	0.371	0.075	−0.150	0.609	−0.073	0.842
GPx I	0.082	0.702	0.082	0.781	−0.723*	0.018
GPx II	0.641**	<0.001	0.583*	0.029	0.401	0.251
ΔGPx	0.416*	0.043	0.348	0.223	0.641*	0.046

NW – volunteers with normal BMI, OB – volunteers with BMI> 35, I – data obtained before the experiment; II – data obtained after 20th cryotherapy;* *p* < 0.05,**p < 0.001

## Discussion

To the best of our knowledge there has been no previous research on groups of young people with second degree or class II obesity (BMI between 35.00 and 39.99). Extreme obese subjects are rarely participants in research studies, mainly due to the coexistence of many diseases and limited mobility [17].

In this study, we found that the level of TOC/TOS before WBC (at baseline) was significantly higher in the OB group compared to the NW group. Similar results were reported by Rowicka [18] in obese children as well as by Molnar et al. [19] and Demircan et al. [20] in adults. In the current literature, there is data that suggests that 20 WBC treatments can cause a decrease in oxidative stress. Such observations were postulated in a report by Lubkowska [21]. In the study, Lubkowska et al found that 20 WBC sessions caused a decrease in conjugated dienes and malonic dialdehyde in healthy men.

Previous studies have shown that obesity is associated with a reduction in TAS/TAC in both children and adults [18,20,22]. Our results showed that there were no significant differences in baseline total antioxidative status/capacity values between the OB and NW groups. Moreover, 20 cryostimulation sessions did not affect its change. We pointed out, however, that the OSI level before WBC was significantly higher in the OB compared to the NW group. Higher OSI in people with obesity has been indicated earlier by Rowicka [18]. In addition, we have shown that 20 WBC sessions didn’t influence the OSI indicator in both groups.

A significantly higher oxidative status in the OB group may be associated with the pathophysiology of obesity [5,6,8,23]. Obesity can induce systemic oxidative stress through various biochemical mechanisms, such as stimulation of peroxide production through NADPH oxidases, oxidative phosphorylation, glycerylaldehyde autoxidation, protein kinase C activation and activation of the polyol and hexosamine pathways [24]. Other factors that also contribute to the increase of oxidative stress in obesity include hyperleptinemia [4,7], chronic inflammation and the production of reactive oxygen species after a meal [25]. The first line of antioxidant defence involves the activity of superoxide dismutase (SOD1), catalase (CAT) and glutathione peroxidase (GPx). Some research data has indicated that activities of blood antioxidant enzymes are lower in obese people and show dependence on higher body fat and central obesity associated directly with the high amount of visceral fat [25–27]. Our results indicate that in the OB group, significantly lower activity of antioxidative enzymes was associated only with SOD1. Although, the baseline CAT activity was similar in both groups, a significant increase after 20 WBC treatments was shown only in the obese group. The differences in the activity of these enzymes between the examined groups were shown by the CAT/SOD1 ratio, which was significantly lower before WBC treatments in NW group. However, we have shown that after 20 WBC sessions, this ratio was similar in both groups. Significant differences in GPx activity between the groups were observed only after 20 treatments. Differences in groups were not significant.

According to Lubkowska et al. [21], changes in the activities of antioxidative enzymes are dependent on the number of cryostimulations. Their study showed that increasing the number of treatments from 10 to 20 in the series led to a significant increase in SOD1 activity in young healthy men. These results are not consistent with the results obtained in our study. We observed a significant decrease in SOD1 activity in the NW group despite the number of sessions being 20. The extended series did not allow for the development of adaptive changes, with a return to the initial measures in this group. However, in the case of participants with obesity, our results coincide with the observations of Lubkowska [21].

Lubkowska et al. [11,21] reported that 20 WBC treatments significantly changed antioxidant system capacity through adaptive changes. Our results showed that 20 WBCs caused an increase in the antioxidant enzymatic potential in erythrocytes in the OB group, without significant change in plasma antioxidative status. According to the literature, these differences could be associated with diet, which affects the amount and quality of low molecular antioxidants that help build the plasma's antioxidant potential [28,29]. Moreover, Dulian et al. [30] reported that there are positive correlations between the concentrations of pro-inflammatory cytokines, such as irisin or other adipokines, and body fat content. Simultaneously, the authors showed a negative correlation between body fat and muscle mass. It has been documented that subcutaneous adipose tissue is a main source of irisin in response to cold conditions [30]. Therefore, in addition to the number of treatments in the series, the next factor for modifying the anti-inflammatory effect and the PAB during response to WBC is fat content, particularly in subcutaneous tissue [30]. These dependencies could cause differences in CAT activity in our results obtained for the OB group, compared to the ones reported previously. Lubkowska et al. [21] reported no changes in CAT in young men (BMI 22.12–33.20 kg/m^2^), but similar results were reported for obese men (BMI 30.39 ± 4.31 kg/m^2^) [31].

Furthermore, we observed significantly lower SOD1 activity in the OB group. This relationship has been earlier reported by Olusi et al. [32] and Ozata et al. [33]. After a series of 20 WBC treatments, SOD1 activity in the OB group increased, while in the NW group it decreased. The changes observed in the NW group confirm previous reports [31]. We indicated that after a series of 20 WBC treatments, SOD1 activity between the groups did not differ significantly. In our work, it was pointed out for the first time that the CAT/SOD1 ratio at baseline (before WBC treatment) in obese patients is observed to be significantly higher than in people with normal BMIs.

Baseline GPx activity was higher in the OB group Ater 20 WBC treatments it slightly increased in this group and compared to NW its was significantly higher. However, in the NW group, a downward trend in GPx activity was demonstrated, which was also observed in young healthy men by Lubkowska et al. [21], while in the OB group there was a statistically insignificant increase in the activity of this enzyme. Conversely, in another study Lubkowska et al. [31] reported that 20 WBC treatments did not induce changes in GPx in men that had an average BMI of 30.39 ± 4.31. It is possible, however, that the higher BMIs of the participants of our study (35.88 ± 0.53) may contribute to this difference. In discussing differences in response to WBC, individual variability, and the influence of varying levels of motor activity across subjects cannot be excluded. Additionally, in our opinion, the impact of the amount and distribution of adipose tissue on WBC response should be investigated in subsequent cryostimulation studies in people with higher classes of obesity.

Significant age differences between the project participants assigned to NW and OB groups were identified. The correlation analysis showed that the relationship with age is weak and concerns only some of the studied variables. Numerous correlations for GPx have been shown. Additionally, it is important because in our project, changes in the activity of this enzyme after a series of treatments were dynamic and multidirectional. The obtained Pearson correlation coefficients indicate the necessity to repeat this test, and the age differences are the basic limiting factor in this study. The relationships observed for the remaining variables were weak, and age differences in this case will not constitute study limitations.

The observed relationships of the activity of antioxidant enzymes with age confirm the previous observations of Inal [34]. These studies, as in our project, showed a negative correlation of SOD activity with age and a positive correlation between age and the activities of CAT and PGx.

For all our subjects, we have found a weak relationship between the change in SOD1 activity and the change in GPx activity. In people with normal BMI values, an inverse correlation was found between TAS/TAC and the level of SOD1 enzyme activity, measured before the start of the treatment cycle in the cryochamber. Such a phenomenon has already been described earlier and the inverse correlation between enzymatic and non-enzymatic antioxidants was noted by Rizvi et al. [35]. These dependencies are part of the compensatory mechanisms aimed at maintaining the balance in the system. No such relationship was observed in the group of obese people, which emphasizes the differentiation of PAB maintenance processes in patients with morbid obesity [3]. In the OB group, a positive correlation was found between the activity of SOD1 and GPx, and between the change in SOD1 activity and the change in GPx activity. As it was stated before, this enzyme (GPx) also showed numerous associations with the age of our subjects (please see Table 3).

In conclusion, it should be emphasized that the human body has various mechanisms that help maintain PAB. Various stimuli, including extremely low temperatures, may modify one or more of these mechanisms. Our results demonstrate that WBC can influence antioxidative enzyme activity (CAT) but does not affect plasma antioxidant potential in people with extreme obesity. In our opinion, the lack of improvement in PAB may result from the insulating effect of adipose tissue, which reduces the penetration of the thermal stimulus into the body. Our results, despite their pilot nature, make us verify the treatment procedures for people with an increased body fat content.

## Conclusions

In this study, while 20 WBC treatments did not influence plasma oxidative and antioxidative status in people with extreme obesity, CAT activity was observed to significantly increase in this group. It is possible that a greater number of treatments are needed for people with BMIs higher than 35 to yield the changes reported for lean individuals. Ambiguous data in the literature shows the need for further research to identify factors that may influence the response of an organism to WBC such as visceral and subcutaneous fat, diet, or cold tolerance predisposition.
